# Significant reduction of physical activity in patients with neuromuscular disease during COVID-19 pandemic: the long-term consequences of quarantine

**DOI:** 10.1007/s00415-020-10064-6

**Published:** 2020-07-13

**Authors:** Vincenzo Di Stefano, Giuseppe Battaglia, Valerio Giustino, Andrea Gagliardo, Michele D’Aleo, Ottavio Giannini, Antonio Palma, Filippo Brighina

**Affiliations:** 1 Department of Biomedicine, Neuroscience and Advanced Diagnostic (BIND), University of Palermo, Via G. La Loggia, 1, 90129 Palermo, Italy; 2 Department of Psychology, Educational Science and Human Movement, University of Palermo, Palermo, Italy

**Keywords:** COVID-19, Coronavirus pandemic, Neuromuscular, Quarantine, Lockdown, Physical activity

## Abstract

**Background:**

Quarantine was the measure taken by governments to control the rapid spread of COVID-19. This restriction resulted in a sudden change in people’s lifestyle, leading to an increase in sedentary behavior and a related decrease in the practice of physical activity (PA). However, in neuromuscular diseases patients need to perform regular PA to counteract the negative consequences of the disease. Hence, the aim of this study was to estimate the levels of PA, measured as energy expenditure (MET–minute/week), among patients with neuromuscular disease (NMD) before and during the last week of quarantine.

**Methods:**

A total of 268 Italian subjects, living in Sicily, completed an adapted version of the IPAQ-SF. Participants comprised 149 NMD, enrolled at the Neuromuscular Clinic of Palermo and 119 healthy subjects (control group). The SF-12 questionnaire was also administered to NMD. The Mann–Whitney *U* and the Kruskal–Wallis rank-sum tests were used for statistical analyses.

**Results:**

We observed a significant decrease of the total weekly PA level during COVID-19 quarantine in both patients and controls. Moreover, a significant difference in the total weekly PA level was found depending on the presence of neuromuscular disease, impaired walking, gender and BMI. Finally, we found a correlation between SF-12 scores and the entity of the reduction of PA level during quarantine, thus confirming a relevant association with the quality of life in NMD.

**Conclusion:**

Our study confirmed that COVID-19 quarantine has affected the practice of PA among both NMD and healthy controls.

## Introduction

The coronavirus disease 2019 (COVID-19) pandemic has grown since late 2019, causing an unprecedented crisis and affecting the lives of millions of people worldwide [1, 2]. Hence, COVID-19 has been declared a public health emergency and in many countries people were asked to live in home-confinement for several months, while hospitals have been forced to reduce their outpatient activities to cope with the high number of hospitalizations [3, 4]. Apart from the well-known symptoms of COVID-19 (fever, diarrhea and respiratory impairment), neurological symptoms are reported in up to one-third of cases [2, 5, 6]. Furthermore, pandemic-associated psychological sequelae have been reported [1, 7]. In fact, the restrictive measures and the quarantine imposed by the governments have contributed to further indirect effects of pandemic by influencing (negatively) many aspects of everyday life, for instance, the practice of PA. As a result of COVID-19 pandemic, many people all around the world have suddenly become inactive and sedentary, with important consequences for both healthy people and patients affected by several kinds of disease [2, 5, 8, 9].

It is a well-known fact that inactivity and sedentarism lead to specific alterations in the skeletal muscle, contributing to insulin resistance, impairment of the oxidative function, and rapid hypotrophy [10–13]. These changes in the muscle pathophysiology usually take place after a couple of weeks, but they can also be faster in older people or patients affected by neuromuscular disease [10, 14].

Neuromuscular diseases affect both adults and children causing a relevant disability in the lifespan [14, 15]. They include disorders of: skeleton muscle (i.e. muscle dystrophies, inflammatory myopathies, muscle channalopathies), neuromuscular junction (i.e. myasthenia gravis, Eaton–Lambert syndrome), and peripheral nerve (i.e. familial amyloid neuropathies, chronic inflammatory demyelinating polyradiculoneuropathy). Moreover, patients with neuromuscular disease (NMD) require strict follow-up, essential immunotherapies, muscle rehabilitation and physical exercise [14–20]. In particular, NMD need an active physical activity (PA), through the practice of regular exercise, to improve muscle strength, endurance ability and to prevent osteoarticular complications from disuse [15, 17, 21]. PA and exercise should be performed under the supervision of a reference specialist and in close collaboration with physiotherapists in an integrated and individualized approach [15, 16, 21]. For these reasons, it is not difficult to hypothesize that COVID-19 pandemic-related Italian government limitations have contributed to a significant change in the care of NMD due to the difficult access to immunosuppressive treatments and physiotherapy [15].

Notwithstanding the relevant impact of COVID-19 pandemic on the health of NMD, scientific task forces have provided recommendations for the care of neuromuscular diseases [3, 4, 15, 17]. However, very little has been discussed about the fundamental role of PA and physiotherapy in this subset of patients during the lockdown [17]. Isolation at home can lead to poor nutrition, low sleep quality, reduced PA levels and sedentary habits with different negative consequences, such as increased body fat, decreased muscle mass, insomnia and depression [1, 2, 10, 22, 23]. Therefore, it is important to highlight these unwanted consequences of quarantine to develop and provide practical and useful recommendations.

In the present cross-sectional study we aimed to explore the impact of COVID-19 lockdown on PA in NMD and to quantify the expected reduction of PA levels, as well its effect on the quality of life.

## Methods

### Study design and procedure

The study design of the research is a cross-sectional survey conducted through a detailed interview. The survey conducted for the study included an adapted version of the International Physical Activity Questionnaire Short-Form (IPAQ-SF) [24, 25] and a Short-Form Health Survey (SF-12) [26]. Before being admitted to the research, all participants signed informant consent. The Ethical Board of the University of Palermo approved the study in conformity with the Declaration of Helsinki principles.

### Participants

Participants, living in Sicily, were recruited between April 20 and May 4, 2020 (during COVID-19 quarantine in Italy). It is well-known that in this period, due to the restrictive measures adopted by the government, all sports facilities were closed and the practice of outdoor PA in public parks and gardens was forbidden [5, 22]. Participants were enrolled at the Neuromuscular Clinic of the University of Palermo regarding patients with neuromuscular diseases (*n* = 149), while healthy controls (*n* = 119) were enrolled among not-affected partners and caregivers.

### Data collection

The data were collected through a questionnaire administered by telephone to the participants. The adapted version of the IPAQ-SF we have chosen allowed us to assess, at the same time, the levels of PA both before and during the last 7 days of COVID-19 quarantine [24, 25]. The levels of PA were measured as energy expenditure (MET–minutes/week).

The adapted version of the IPAQ-SF comprised 31 questions assessing frequencies and durations of each PA intensity, i.e. sitting, walking, moderate-intensity physical activities, and vigorous-intensity physical activities. In particular, the questionnaire included questions about: demographic and anthropometric data; PA before quarantine; type of work done during quarantine; type of house where lived during quarantine; vigorous-intensity PA before and during quarantine, moderate-intensity PA before and during quarantine, walking activities before and during quarantine, sitting activities before and during quarantine; information concerning the practice of PA in home setting during quarantine [25].

Finally, a Short-Form Health Survey (SF-12) was administered to the NMD [26]. This questionnaire consisted of two assessment domains: the physical health component score (PCS) and the mental health component score (MCS).

### Scoring protocol

As for the IPAQ-SF adapted version, we considered the total PA level and the moderate-to-vigorous PA (MVPA) level for both before and during quarantine. Furthermore, we analyzed both parameters in relation to the gender, age, and BMI variables. The weekly PA level of both considered parameters were calculated as energy expenditure in MET–minutes/week (MET–min/wk) [27]. We used the corresponding metabolic equivalent task (MET) assigned to each type of PA (i.e. 3.3, 4.0 and 8.0 for walking, moderate-intensity physical activities and vigorous-intensity physical activities, respectively) to estimate the weekly level of energy expenditure. Afterwards, we computed the sum of energy expenditure of walking, moderate-intensity physical activities, and vigorous-intensity physical activities in MET–min/wk for the “total PA” level and the sum of moderate-intensity physical activities, and vigorous-intensity physical activities in MET–min/wk for the “MVPA” level [25, 28]. Hence, we multiplied the corresponding MET basal level for each type of PA per minutes of practice during the week to calculate the MET weekly level (https://www.ipaq.ki.se) [25, 28].

The distribution of the PA level difference between before and during quarantine was calculated for each different PA intensity and for the total PA level (i.e. the sum of walking, moderate-intensity physical activities, and vigorous-intensity physical activities) in walking subjects. In fact, in patients with impaired walking ability, we did not calculate the total PA level, but, for these subjects, we considered instead the MVPA level (i.e. the sum of moderate-intensity physical activities and vigorous-intensity physical activities), which has been often used in elderly (https://www.ipaq.ki.se) [29, 30].

As regards the SF-12, reference values were published for both healthy subjects and patients with neurological diseases for the Italian population [31, 32]. PCS and MCS scores range from 0 to 100, with higher scores indicating a better health-related quality of life [26, 33].

### Statistical analysis

Percentages were calculated to describe the categorical variables and we reported continuous variables as means with standard deviation. We compared categorical variables between groups using the Chi-square test and continuous variables through the Mann–Whitney test. Percentiles, means, and standard deviations were calculated to represent the PA level for the categorical variables. For statistical analysis, we used the labels “MET pre COVID-19” and “MET during COVID-19” to represent the PA level before and during quarantine, respectively; “∆MET” to indicate the PA level difference between before and during quarantine. Box-plots were used to graphically represent the quantitative variables. The Mann–Whitney *U* test for continuous variables was chosen to compare the distribution of the total weekly PA level before and during quarantine. Subsequently, we analyzed the relationship between the parameters “MET pre COVID-19” and “MET during COVID-19” and the gender, age, and BMI variables through a bivariate analysis. In particular, we used the Kruskal–Wallis rank-sum test for the abovementioned variables (i.e. age, gender, BMI), SF-12 scores and disease subtype in NMD. Pearson’s correlation coefficient was calculated between SF-12 scores and ∆MET parameters and MVPA. We performed all tests using SPSS Statistic (v26) and established the level of significance at < 0.05.

## Results

### Study population

A total of 268 Italian subjects, all from Sicily Region, both physically active and inactive, completed the questionnaire. Among these, 149 were affected by a neuromuscular disease and they were enrolled at the Neuromuscular Clinic of Palermo, while 119 healthy subjects were recruited as control group.

Among the participants of the group with neuromuscular disease (62% males), 119 patients (80%) were able to walk independently and 30 patients (20%) showed impaired walking ability. Regarding the type of disease, 19 patients (13%) had an acquired or hereditary myopathy (MY); 69 patients (46%) presented a diagnosis of acquired or hereditary polyneuropathy (PN); 49 patients (33%) suffered from a disorder of the neuromuscular junction (NJD); and the last group consisted of 12 patients (8%) with a genetically confirmed degenerative disease (ND, i.e. hereditary spastic paraplegia, spinal muscular amyotrophy).

There were no significant differences in age and gender between patients and healthy controls (Table 1). Of interest, NMD showed significantly higher BMI scores (*p* = 0.044), although this difference resulted no significant when only walking patients were considered.

**Table 1:** Demographics and MET levels in patients with neuromuscular disease and healthy controls

	NMD	Healthy controls	*p*
Age (years)	57.3 ± 13.7	56 ± 6.8	0.2
Gender (males)	93/149 (62%)	74/119 (62%)	0.93
BMI (kg/m^2^)	27.26 ± 4.4	26.3 ± 2.4	0.044*
MET-vigorous pre COVID-19 (min/wk)	70.1 ± 361.9	2081.8 ± 4945.3	< 0.0001***
MET-vigorous during COVID-19 (min/wk)	37.1 ± 303.9	861.9 ± 1662.9	< 0.0001***
∆MET-vigorous (min/wk)	− 33 ± 219.2	− 1219.9 ± 4920.8	< 0.0001***
MET-moderate pre COVID-19 (min/wk)	263.2 ± 606.9	1153.3 ± 2424.6	< 0.0001***
MET-moderate during COVID-19 (min/wk)	146.9 ± 450.6	925.4 ± 3675.6	< 0.0001***
∆MET-moderate (min/wk)	− 116.2 ± 323.2	− 227.87 ± 4076.9	0.47
MET-walking pre COVID-19 (min/wk)	547.7 ± 733.2ª	1271.5 ± 2703.6	0.028*
MET-walking during COVID-19 (min/wk)	211.9 ± 534 ª	574.9 ± 1731.3	0.006**
∆MET-walking (min/wk)	− 335.8 ± 524.3ª	− 696.5 ± 3167.1	0.6
MET-total pre COVID-19 (min/wk)	901.3 ± 1299.6ª	4506.5 ± 7600.1	< 0.0001***
MET-total during COVID-19 (min/wk)	400.6 ± 1088.5ª	2362.3 ± 4498.9	< 0.0001***
∆ MET-total (min/wk)	− 500.7 ± 705.7ª	− 2144.3 ± 8630.7	0.006**
MPVA pre COVID-19 (min/wk)	333.3 ± 483.8	3235.7 ± 3684.7	< 0.0001***
MPVA during COVID-19 (min/wk)	184 ± 440.3	1787.3 ± 2669.3	< 0.0001***
∆MET-MPVA (min/wk)	− 149.3 ± 426.8	− 1447.8 ± 7611.3	0.006**

*NMD* patients with neuromuscular disease

ªcalculated only in NMD with preserved walking

^*^Mann–Whitney *U* test, *p* < 0.05

^**^Mann–Whitney *U* test, *p* < 0.01

^***^Mann–Whitney *U* test, *p* < 0.001

### Physical activity level

The distribution of ∆ MET was calculated for different levels of PA intensity (i.e. vigorous-intensity PA; moderate-intensity PA; walking activity) in all subjects. The Mann–Whitney *U* test showed a significant difference in the distribution of each intensity of PA and in both parameters which we have considered for the PA level (i.e. the total PA level parameter and the MVPA level) in the before-quarantine condition as well as during quarantine condition in NMD compared to healthy subjects (Table 1).

In healthy controls, a significant reduction of PA was reported during quarantine compared to before quarantine for vigorous-intensity PA (*p* = 0.04), moderate-intensity PA (*p* = 0.01), walking activity (*p* < 0.0001), total PA level (*p* < 0.0001) and MVPA level (*p* = 0.001). In NMD, a significant reduction of PA was reported for walking activity (*p* < 0.0001), total PA level (*p* < 0.0001) and MVPA level (*p* = 0.04), while no difference was found for vigorous-intensity PA (*p* = 0.69) and moderate-intensity PA (*p* = 0.07), thus explaining the reason why in NMD basal levels of energy expenditure start from very low values.

Moreover, we estimated the impact of quarantine on each PA intensity, in the total PA level parameter and in the MVPA level calculating the difference between energy expenditure during and before quarantine (∆MET) finding a significant difference in vigorous-intensity PA (*p* < 0.0001), in total PA level parameter (*p* = 0.006) and in the MVPA parameter (*p* = 0.006). We did not find any significant difference in moderate-intensity PA and walking activity.

**Fig. 1:**
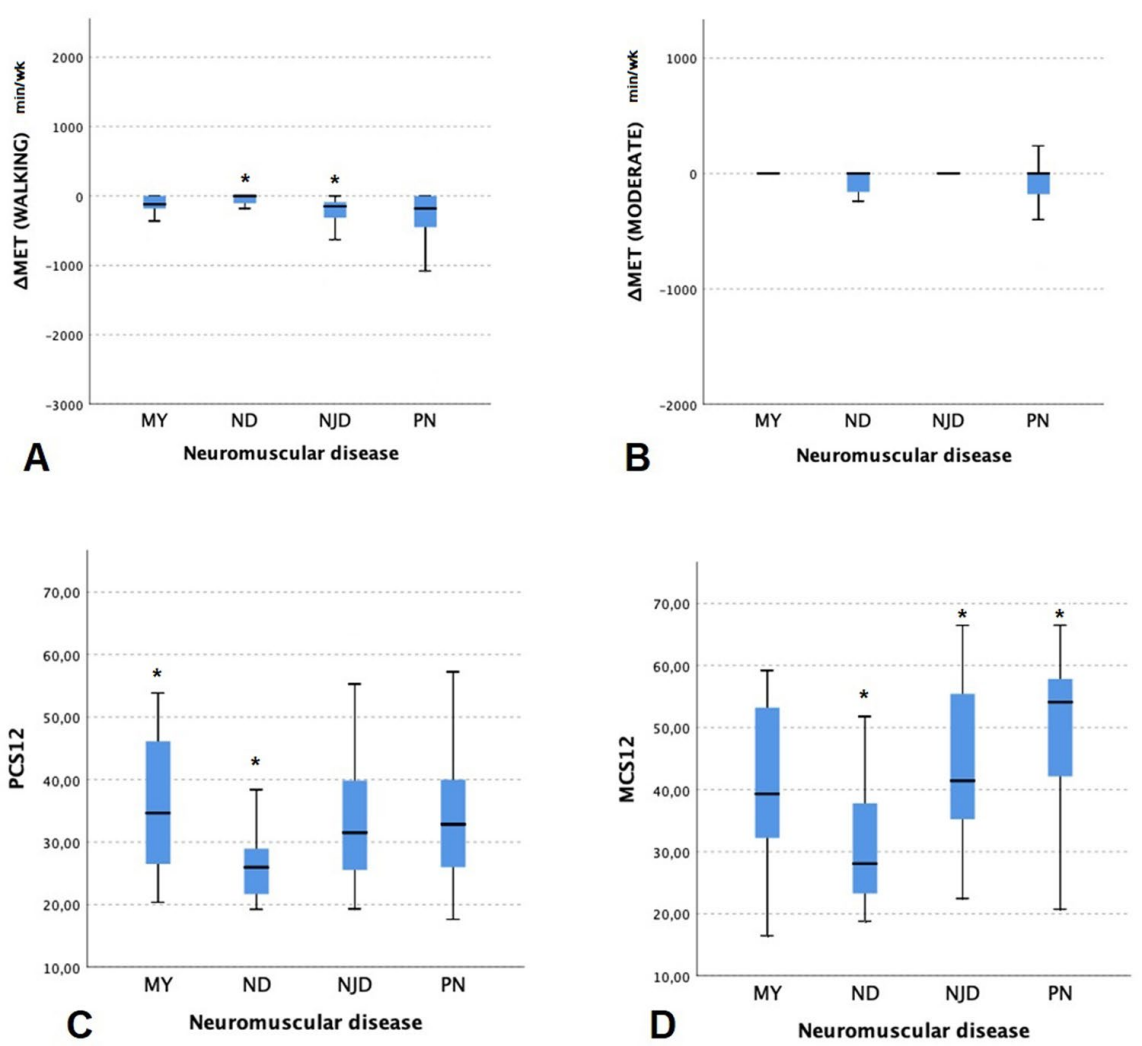
The figure shows the entity of MET reduction for walking (**a**) and moderate energy expenditure (**b**) and SF-12 scores (**c**, **d**) depending on the disease subtype. *MY* acquired or hereditary myopathy, *ND* genetically-confirmed degenerative disease, *NJD* disorder of the neuromuscular junction, *PN* acquired or hereditary polyneuropathy, *PCS-12* physical health component score of SF-12, *MCS-12* mental health component score of SF-12. *p < 0.05

∆MET measures (∆MET-Vigorous, ∆MET-Moderate and ∆MET-MVPA) were not differently distributed in relation to impaired walking, gender, age, and BMI among NMD. Moreover, we found no significant difference in relation to the disease subtype, except for ∆ MET-Walking that was higher in patients with neuromuscular junction disorder than patients of the neurodegenerative subgroup (*p* = 0.048, Fig. 1).

### SF-12

NMD showed reduced scores for both PCS-12 (33.7 ± 10.1) and MCS-12 (45.2 ± 13.0) domains compared to Italian general population. SF-12 scores were not differently distributed in relation to age. Of interest, among NMD both PCS-12 and MCS-12 scores were significantly lower in patients with impaired walking (*p* < 0.0001 and *p* = 0.011, respectively); men showed reduced MCS-12 scores (*p* = 0.008) and PCS-12 scores were significantly lower in patients with higher BMI (*p* = 0.036). The Kruskal–Wallis test reported a significant difference for both PCS-12 and MCS-12 in relation to the neuromuscular disease (*p* = 0.029 and *p* < 0.0001, respectively, Fig. 1). In particular, we found lower PCS-12 scores in ND patients when compared to MY patients (*p* = 0.026); furthermore, ND patients showed lower MCS-12 scores compared to NJD and PN patients (*p* = 0.023 and *p* < 0.0001, respectively).

A linear correlation was found between PCS-12 scores and both ∆ MET total (− 0.276, *p* = 0.002) and MVPA (− 0.229, *p* = 0.005). Moreover, we found a correlation between MCS-12 scores and ∆MET total (− 0.192, *p* = 0.036).

## Discussion

In this study we investigated the impact of the COVID-19 quarantine on PA levels and quality of life in NMD.

During these last months, the quarantine has allowed to restrain the rapid spread of COVID-19 [3]. However, this containment measure may have had side effects on the health of the populations [1, 2]. Indeed, quarantine has implied a sudden change in people’s lifestyle, leading to an increase in sedentary behavior [10, 25, 34]. Such a radical change in daily life can have to negative effects in high-risk patients, who need to perform regular exercise to counteract the negative consequences of certain disease, such as neuromuscular diseases [21].

Our understandings on the changes inducted in the skeleton muscle by inactivity come from several models including bed rest, limb suspension and step reduction [10, 11, 35]. Data from studies on “bed rest” established that muscle atrophy, especially in antigravity muscles, appears very soon after only two days of inactivity [10, 35]. A prolonged immobilization can induce a significant reduction of protein synthesis in the muscle fibers thus conducting to muscle mass loss [12, 13, 35]. Of note, it has been recently hypothesized that physical inactivity may also cause damage at the neuromuscular junction with muscle denervation [10]. Finally, it has to be considered that a more sensible muscle mass loss is reported following physical inactivity in older people and in neuromuscular disease, compared to healthy young subjects [10, 13]. Hence, there is some concern about the consequences of physical inactivity especially in NMD and older people.

Previous studies have recently pointed out the reduction of PA levels in the general population during COVID-19 pandemic [8, 22]. In the scientific literature there are a few studies that have explored this topic in healthy subjects or in athletes [22, 25, 34], however, no studies have examined the consequences of quarantine in NMD.

As expected, a significant reduction in PA levels during the pandemic in both NMD and healthy controls was demonstrated. Moreover, in controls subjects was found a significant reduction in all PA parameters considered, while, NMD showed a significant decrease in walking activity, total weekly PA level and weekly MVPA level. These results reflect the fact that moderate-intensity PA and walking activities were similarly affected in both patients and controls, while vigorous-intensity PA seem to be more reduced in controls. This was in agreement with a recent study that reported a high level of total weekly energy expenditure before the COVID-19 quarantine in healthy subjects [25]. Vigorous-intensity and moderate-intensity PA, which require a suitable state of health to perform certain physical efforts represented a relevant part of baseline PA levels in healthy controls, but not in NMD. Anyhow, the quarantine has negatively influenced walking activities above all in NMD. The key explanation for this result could be related to the fact that walking represents a low intensity and aerobic activity characterized by easy accessibility for all the population [36].

There is evidence that PA improves both mental and physical health [8, 21, 34]. Therefore, it is reasonable to hypothesize that a decrease in PA levels could have an impact on both health domains, as confirmed by the association between SF-12 scores and the extent of PA reduction that we have found. Surprisingly, both PCS-12 and MCS-12 scores correlated with ∆MET-Total and, moreover, PCS-12 correlated with MVPA level. Furthermore, compared to Italian population [31, 32], NMD showed reduced SF-12 scores, especially patients with impaired walking. In particular, the mental health component resulted more affected among men, while the physical component was more altered in patients with higher BMI. Finally, specific alterations resulted from different diseases: ND patients showed lower PCS-12 scores than MY patients and lower MCS-12 scores compared to NJD and PN patients. These results were also expected, as ND patients (SMA, HSP) usually have a more significant disability level than other forms of neuromuscular diseases [14].

In conclusion, this study highlights the negative impact of COVID-19 quarantine on PA levels in NMD and healthy subjects. A significant reduction in PA has been reported in NMD, especially walking activities. Moreover, the extent of PA reduction was related to the perceived physical and mental health of NMD, thus influencing their quality of life. Sedentary behaviors can have negative consequences on the health of the entire population, in particular for those with additional risk factors and neuromuscular diseases. Therefore, since outdoor activities are not practicable due to the quarantine, it is essential to maintain an active lifestyle by performing exercise in a home-based setting for healthy subjects as well as for patients. Indeed, PA and physiotherapy are fundamental for NMD to avoid further loss of muscle mass and to slow the progression of the disease.

### Limitations

This is a cross-sectional survey exploring the effect of COVID-quarantine in the PA levels of NMD. Our results come from comparison of the two condition, “pre” and “during” quarantine; however, the evaluation of the two conditions comes from the same interview and this may have biased the collection of data. PA levels are quite easy to recall in memory and should not be affected by the mental perception, at difference with psychological variables that may be affected by memory. For this reason, we administered SF-12 only during quarantine renouncing to a comparison with the before quarantine status. Future studies are needed to clarify whether differences in PA levels could affect other domains of health and life in healthy subjects and in NMD. Finally, we did not report data on follow-up. Future studies monitoring PA levels for several months after lockdown could offer new interesting clues to the mechanisms of recovering after muscle mass loss and hypotrophy and provide more useful information to predict outcome and response to different rehabilitation strategies.

## References

[1] Troyer EA, Kohn JN, Hong S (2020). Are we facing a crashing wave of neuropsychiatric sequelae of COVID-19? Neuropsychiatric symptoms and potential immunologic mechanisms. Brain Behav Immun.

[2] Velavan TP, Meyer CG (2020). The COVID-19 epidemic. Trop Med Int Health.

[3] Bersano A, Pantoni L (2020). On being a neurologist in Italy at the time of the COVID-19 outbreak. Neurology.

[4] World Health Organization (2020). Q&A on coronaviruses (COVID-19).

[5] de Seze J, Lebrun-Frenay C (2020). Covid-19, the pandemic war: implication for neurologists. Rev Neurol.

[6] Manji H, Carr AS, Brownlee WJ, Lunn MP (2020). Neurology in the time of COVID-19. J Neurol Neurosurg Psychiatry.

[7] Maugeri G, Castrogiovanni P, Battaglia G, Pippi R, D’Agata V, Palma A (2020). The impact of physical activity on psychological health during Covid-19 pandemic in Italy. Heliyon.

[8] Chen P, Mao L, Nassis GP, Harmer P, Ainsworth BE, Li F (2020). Coronavirus disease (COVID-19): the need to maintain regular physical activity while taking precautions. J Sport Health Sci.

[9] Katzmarzyk PT, Church TS, Craig CL, Bouchard C (2009). Sitting time and mortality from all causes, cardiovascular disease, and cancer. Med Sci Sports Exerc.

[10] Narici M, De Vito G, Franchi M, Paoli A, Moro T, Marcolin G (2020). Impact of sedentarism due to the COVID-19 home confinement on neuromuscular, cardiovascular and metabolic health: physiological and pathophysiological implications and recommendations for physical and nutritional countermeasures. Eur J Sport Sci.

[11] Mikines KJ, Richter EA, Dela F, Galbo H (1991). Seven days of bed rest decrease insulin action on glucose uptake in leg and whole body. J Appl Physiol.

[12] Mortensen B, Friedrichsen M, Andersen NR, Alibegovic AC, Højbjerre L, Sonne MP (2014). Physical inactivity affects skeletal muscle insulin signaling in a birth weight-dependent manner. J Diabetes Complicat.

[13] Paddon-Jones D, Sheffield-Moore M, Cree MG, Hewlings SJ, Aarsland A, Wolfe RR (2006). Atrophy and impaired muscle protein synthesis during prolonged inactivity and stress. J Clin Endocrinol Metab.

[14] Butterfield RJ, Johnson NE (2016). Neuromuscular disease. J Pediatr Rehabil Med.

[15] Guidon AC, Amato AA (2020). COVID-19 and neuromuscular disorders. Neurology.

[16] Westerberg E, Molin CJ, Lindblad I, Emtner M, Punga AR (2017). Physical exercise in myasthenia gravis is safe and improves neuromuscular parameters and physical performance-based measures: a pilot study. Muscle Nerve.

[17] Solé G, Salort-Campana E, Pereon Y, Stojkovic T, Wahbi K, Cintas P (2020). Guidance for the care of neuromuscular patients during the COVID-19 pandemic outbreak from the French Rare Health Care for Neuromuscular Diseases Network. Rev Neurol (Paris).

[18] Rajabally YA, Goedee HS, Attarian S, Hartung H (2020) Management challenges for chronic dysimmune neuropathies during the COVID‐19 pandemic. Muscle Nerve. 10.1002/mus.26896 10.1002/mus.26896 PMC7264511 32311114

[19] Di Stefano V, Lupica A, Rispoli MG, Di Muzio A, Brighina F, Rodolico C (2020). Rituximab in AChR subtype of myasthenia gravis: systematic review. J Neurol Neurosurg Psychiatry.

[20] Siciliano G, Schirinzi E, Simoncini C, Ricci G (2019). Exercise therapy in muscle diseases: open issues and future perspectives. Acta Myol.

[21] Anziska Y, Inan S (2014). Exercise in neuromuscular disease. Semin Neurol.

[22] Jukic I, Calleja-González J, Cos F, Cuzzolin F, Olmo J, Terrados N (2020). Strategies and solutions for team sports athletes in isolation due to COVID-19. Sports.

[23] Battaglia G, Paoli A, Bellafiore M, Bianco A, Palma A (2014). Influence of a sport-specific training background on vertical jumping and throwing performance in young female basketball and volleyball players. J Sports Med Phys Fitness.

[24] Craig CL, Marshall AL, Sjöström M, Bauman AE, Booth ML, Ainsworth BE (2003). International physical activity questionnaire: 12-Country reliability and validity. Med Sci Sports Exerc.

[25] Giustino V, Parroco AM, Gennaro A, Musumeci G, Palma A, Battaglia G (2020). Physical activity levels and related energy expenditure during COVID-19 quarantine among the sicilian active population: a cross-sectional online survey study.

[26] Ware JE, Kosinski M, Keller SD (1996). A 12-Item short-form health survey: construction of scales and preliminary tests of reliability and validity. Med Care.

[27] Byrne NM, Hills AP, Hunter GR, Weinsier RL, Schutz Y (2005). Metabolic equivalent: one size does not fit all. J Appl Physiol.

[28] Ainsworth BE, Haskell WL, Leon AS, Jacobs DR, Montoye HJ, Sallis JF (1993). Compendium of physical activities: classification of energy costs of human physical activities. Med Sci Sports Exerc.

[29] Ryan DJ, Wullems JA, Stebbings GK, Morse CI, Stewart CE, Onambele-Pearson GL (2018). Reliability and validity of the international physical activity questionnaire compared to calibrated accelerometer cut-off points in the quantification of sedentary behaviour and physical activity in older adults. PLoS One.

[30] Rääsk T, Maëstu J, Lätt E, Jürimäe J, Jürimäe T, Vainik U (2017). Comparison of IPAQ-SF and two other physical activity questionnaires with accelerometer in adolescent boys. PLoS One.

[31] Vilagut G, Forero CG, Pinto-Meza A, Haro JM, De Graaf R, Bruffaerts R (2013). The mental component of the short-form 12 health survey (SF-12) as a measure of depressive disorders in the general population: results with three alternative scoring methods. Value Health.

[32] Prisnie JC, Sajobi TT, Wang M, Patten SB, Fiest KM, Bulloch AGM (2018). Effects of depression and anxiety on quality of life in five common neurological disorders. Gen Hosp Psychiatry.

[33] Hagell P, Westergren A, Årestedt K (2017). Beware of the origin of numbers: standard scoring of the SF-12 and SF-36 summary measures distorts measurement and score interpretations. Res Nurs Health.

[34] Jiménez-Pavón D, Carbonell-Baeza A, Lavie CJ (2020) Physical exercise as therapy to fight against the mental and physical consequences of COVID-19 quarantine: special focus in older people. Progress Cardiovasc Dis. 10.1016/j.pcad.2020.03.009 10.1016/j.pcad.2020.03.009 PMC7118448 32220590

[35] Kilroe SP, Fulford J, Jackman SR, Van Loon LJC, Wall BT (2020). Temporal muscle-specific disuse atrophy during one week of leg immobilization. Med Sci Sports Exerc.

[36] Battaglia G, Giustino V, Messina G, Faraone M, Brusa J, Bordonali A (2020). Walking in natural environments as geriatrician’s recommendation for fall prevention: preliminary outcomes from the “passiata day” model. Sustainability.

